# The E3-ligase Siah2 activates mitochondrial quality control in neurons to maintain energy metabolism during ischemic brain tolerance

**DOI:** 10.1038/s41419-025-07339-z

**Published:** 2025-01-28

**Authors:** Maria Josè Sisalli, Elena D’Apolito, Ornella Cuomo, Giovanna Lombardi, Michele Tufano, Lucio Annunziato, Antonella Scorziello

**Affiliations:** 1 Division of Pharmacology, Department of Neuroscience, School of Medicine, University of Naples “Federico II”, Naples, Italy; 2 IRCCS Synlab SDN S.p.A, Via Gianturco 113, Naples, Italy

**Keywords:** Cellular neuroscience, Immunochemistry

## Abstract

Mitochondrial quality control is crucial for the homeostasis of the mitochondrial network. The balance between mitophagy and biogenesis is needed to reduce cerebral ischemia-induced cell death. Ischemic preconditioning (IPC) represents an adaptation mechanism of CNS that increases tolerance to lethal cerebral ischemia. It has been demonstrated that hypoxia-induced Seven in absentia Homolog 2 (Siah2) E3-ligase activation influences mitochondrial dynamics promoting the degradation of mitochondrial proteins. Therefore, in the present study, we investigated the role of Siah2 in the IPC-induced neuroprotection in in vitro and in vivo models of IPC. To this aim, cortical neurons were exposed to 30-min oxygen and glucose deprivation (OGD, sublethal insult) followed by 3 h OGD plus reoxygenation (lethal insult). Our results revealed that the mitochondrial depolarization induced by hypoxia activates Siah2 at the mitochondrial level and increases LC3-II protein expression, a marker of mitophagy, an effect counteracted by the reoxygenation phase. By contrast, hypoxia reduced the expression of peroxisome proliferator-activated receptor gamma coactivator 1-alpha (PGC-1α), a marker of mitochondrial biogenesis, whereas its expression was increased after reoxygenation thus improving mitochondrial membrane potential, mitochondrial calcium content, and mitochondrial morphology, hence leading to neuroprotection in IPC. Furthermore, Siah2 silencing confirmed these results. Collectively, these findings indicate that the balance between mitophagy and mitochondrial biogenesis, due to the activation of the Siah2-E3-ligase, might play a role in IPC-induced neuroprotection.

## Introduction

Ischemic preconditioning (IPC), consists of a sublethal ischemic insult that makes the tissue more resistant to subsequent and potentially lethal ischemia [1–4]. This adaptive cytoprotective mechanism represents a fundamental property of living cells and is finely regulated by intracellular pathways involved in the establishment of ischemic tolerance [5–8]. However, there are some aspects of the intracellular mechanisms underlying this phenomenon that still need to be investigated. In this regard, mitochondria represent a fascinating target due to their central role in maintaining neuronal energy homeostasis in physiological and pathological conditions. Previous experiments performed in our and other laboratories demonstrated the key role of mitochondrial dysfunction in the pathogenesis of ischemic stroke [9–13]. Indeed, in addition to their involvement in energy production [14, 15], mitochondria contribute to the balancing of intracellular Ca^2+^ homeostasis within the cytosol and the other cellular compartments, accomplished thanks to the activity of specific channels and transporters localized on the inner and the outer mitochondrial membrane, that allow Ca^2+^ ions to move from the cytosol to the mitochondrial matrix and from the matrix to the cytosol, to the Endoplasmic Reticulum and to the lysosomes [16]. The maintenance of Ca^2+^ within physiological concentration is extremely important for mitochondrial morphology and functional properties such as electron transport and ATP production [17–19]. An alteration of this balance, as it occurs during hypoxia, leads to reactive oxygen species (ROS) generation, mitochondrial membrane depolarization, inflammasome activation, stimulation of mitochondrial dynamics, and apoptotic cell death [20–26]. In this scenario, mitochondrial quality control became crucial for the recovery of homeostatic conditions and the mitochondrial network. Therefore, a constant balance between mitochondrial fission/fusion as well as mitophagy and biogenesis is needed to preserve cell survival in stress conditions, since a disruption of molecular mechanisms involved in mitochondrial quality control contributes to the pathogenesis of ischemia-reperfusion-induced cell death in the brain [27, 28]. Furthermore, preserving mitochondrial function is relevant for preconditioning-induced neuroprotection [29]. In fact, preconditioning positively affects the integrity of mitochondrial oxidative phosphorylation after cerebral ischemia, prevents mitochondrial swelling, protects mitochondrial energy metabolism during cerebral ischemia by avoiding ATP consumption, and increases Mn-SOD expression and activity through the NO/Ras/ERK1-2 pathway [30–33]. Interestingly, in cortical neurons, the neuroprotection elicited by IPC occurs through NO/PI3K/Akt pathway, activates the sodium-calcium exchanger (NCX) isoform 1 and 3, and promotes ER refilling and mitochondrial calcium extrusion, thus preventing intracellular calcium dysregulation induced by OGD [34]. Finally, the demonstration that the tight regulation of both ER and lysosomal Ca^2+^-filling state follows in IPC-induced tolerance in neurons exposed to OGD Reoxygenation (OGD/REOXY) [35] further supports the hypothesis that neuroprotection exerted by ischemic tolerance occurs through a complex and dynamic regulation of the organelles to modulate Ca^2+^ homeostasis. These findings are particularly relevant considering that mitophagy, the key mechanism of mitochondrial quality control, represents a protective strategy to selectively eliminate damaged mitochondria by lysosomes via autophagy in response to diverse stimuli, including hypoxia [27, 28]. Although the molecular mechanisms involved in mitophagy are mainly controlled by the interaction between PINK1 (PTEN-induced putative kinase 1), a mitochondrial serine/threonine kinase, and Parkin (PARK2), a cytosolic E3-ubiquitin ligase [36, 37] triggered by mitochondrial membrane depolarization, many hypotheses are still under investigation. Among them, it has been proposed that the E3-ligase Siah1/2 (seven in absentia homolog-1/2), recruited on mitochondria after depolarization and able to ubiquitinate mitochondrial proteins, activates mitophagy in different pathological conditions like Parkinson’s Disease and cerebral ischemia [38, 39]. Specifically, in cortical neurons Siah2, upon its activation during hypoxia, interacts with the mitochondrial protein AKAP121, NCX3, and VDAC, compromising mitochondrial function [13, 40] and morphology, i.e., fusion and fission, in the subsequent reoxygenation phase [18]. On the other hand, a tight relationship between mitochondrial fission, fusion, and mitophagy has been clearly demonstrated [41]. However, it is extremely interesting to observe that, the disruption in the ATP/ADP ratio occurring during the ischemic stroke [19], correlates with AMP-activated kinase (AMPK) activation and the consequent regulation of mitochondrial biogenesis by phosphorylating and activating the transcriptional factor peroxisome proliferator-activated receptor gamma coactivator 1-alpha (PGC-1α) [42]. Mitochondrial biogenesis represents the counterbalance of mitophagy and is necessary to produce the contingent requirements of ATP as well as for the formation and maintenance of synapses in neuronal stress conditions [43]. It consists of an increase in the mtDNA mass due to the transcription of thirteen key enzymes essential for oxidative phosphorylation that is finely tuned by PGC-1α, whose levels are highly expressed in neuronal cells due to their extreme energy demand, to form a stable complex with NRF1/2 that in turns control TFAM expression [44–46]. More recently, it has been demonstrated a direct interaction between Siah2 and NRF1 [47].

In the present study we explored the hypothesis that ischemia-induced Siah2-E3-ligase might play a neuroprotective effect in ischemic brain preconditioning through the regulation of the mitophagic machinery and the subsequent stimulation of mitochondrial biogenesis.

## Results

### Mitochondrial depolarization promotes Siah2 localization on mitochondria and stimulates mitophagy in cortical neurons

**Fig. 1:**
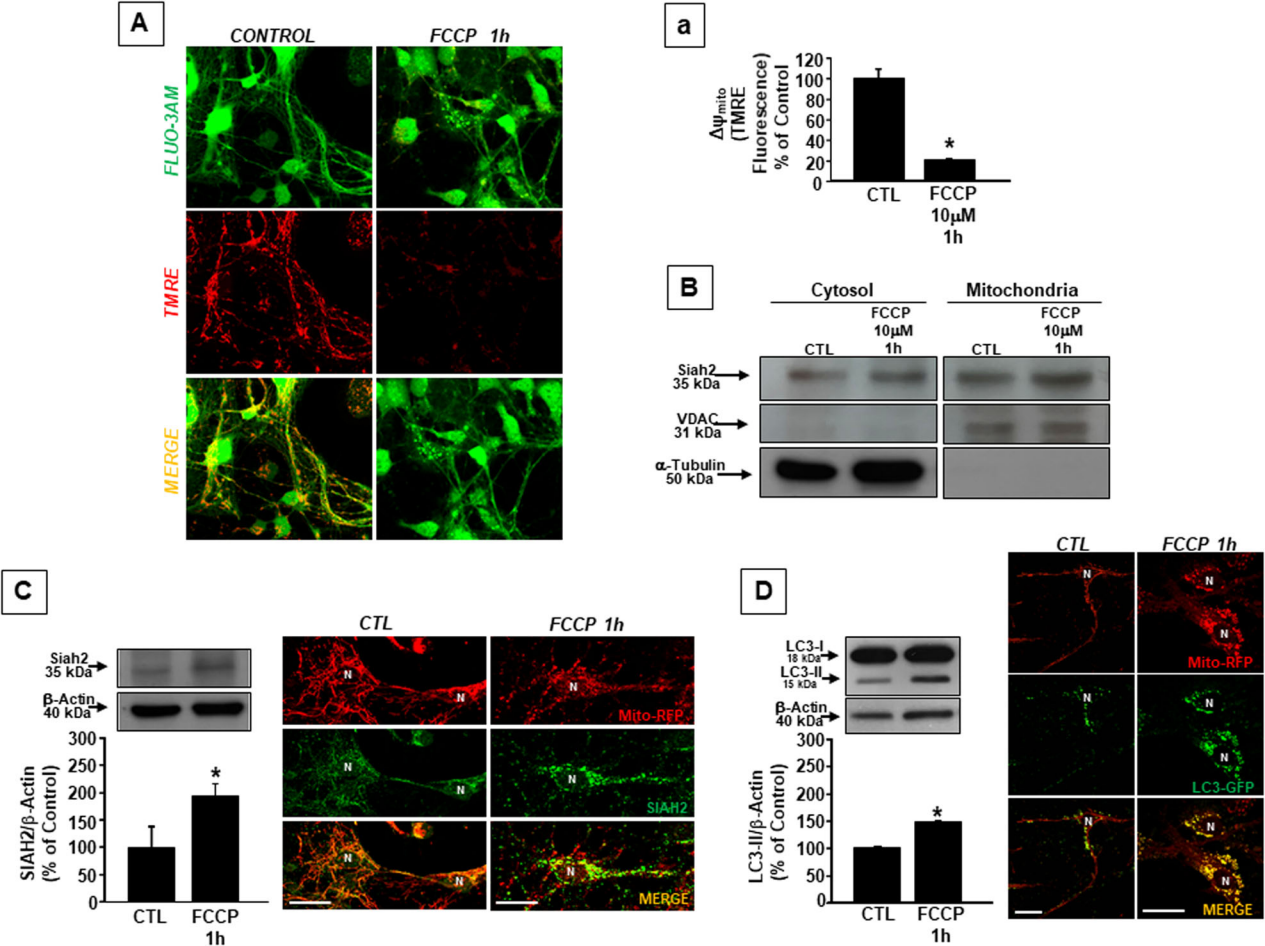
Siah2 redistribution on FCCP-depolarized mitochondria. **A** ΔΨ_m_ in cortical neurons treated with 10 μM FCCP for 1 h and **a** its relative quantification. ΔΨ_m_ was measured by using the fluorescent probe TMRE and confocal microscopy. The reduction of the intensity of TMRE fluorescence (red) is indicative of mitochondria depolarization. The green fluorescent signal corresponds to cells loaded with Fluo4-AM used to identify neuronal morphology in control and FCCP treatment conditions. **a** Quantification of the results reported in **A**; each bar represents the mean±S.E.M of the percentage of different experimental values obtained in three independent experimental sessions. *P < 0.05 vs CTL. **B** Siah2 expression was measured by western blotting in, cytosolic, and mitochondrial fractions of cortical neurons exposed to 1 h FCCP. Immunoblotting for VDAC and Tubulin was used to define the identity of mitochondrial and cytosolic compartments, respectively. **C** Left panel: Siah2 protein expression in cortical neurons after FCCP treatment. Each bar represents the mean±S.E.M of the percentage of different experimental values obtained in three independent experimental sessions. *P < 0.05 vs CTL. Right panel: representative cortical neuron double-labeled with Siah2 (green) and Mito-RFP (red). Siah2/Mito-RFP colocalization was 100% in CTL conditions and was up to 133% ± 1 after FCCP treatment. **D** Left panel: LC3-II protein expression in cortical neurons after FCCP treatment. Each bar represents the mean±S.E.M of the percentage of different experimental values obtained in three independent experimental sessions. Right panel: representative cortical neuron double-labeled with LC3-GFP (green) and Mito-RFP (red). LC3-GFP/Mito-RFP colocalization was 100% in CTL conditions and was up to 154% ± 1 after FCCP treatment. Bars 10 μM.

To demonstrate the role of Siah2 in the mitochondrial quality control mechanism, cortical neurons were treated with the mitochondrial uncoupler carbonyl cyanide p-trifluoromethoxyphenylhydrazone (FCCP) at the concentration of 10 μM for 1 h to induce mitochondrial depolarization and Siah2 activation. As shown in Fig. 1A, the treatment with FCCP induces a significant reduction of mitochondrial membrane potential (ΔΨ_m_), an effect associated with the increase in the expression of Siah2 in the mitochondrial fraction (Fig. 1B). Moreover, mitochondrial depolarization causes an increase in the conversion of Microtubule-associated protein 1 A/1B-light chain 3 (LC3) from its cytosolic form LC3-I into its autophagy-related form LC3-II (Fig. 1D). Interestingly, immunocytochemistry experiments demonstrated both the presence of Siah2 on FCCP-treated mitochondria (Fig. 1C) and the increased colocalization of LC3-GFP on mitochondria stained with mitochondrial targeted-RFP (Mito-RFP) (Fig. 1D), thus confirming a relationship between mitochondrial depolarization and Siah2-mediated mitophagy activation, as mechanism responsible for the activation of mitochondrial quality control.

### Oxygen and glucose deprivation activates Siah2-mediated mitophagy and stimulates mitochondrial biogenesis during the reoxygenation in cortical neurons

**Fig. 2:**
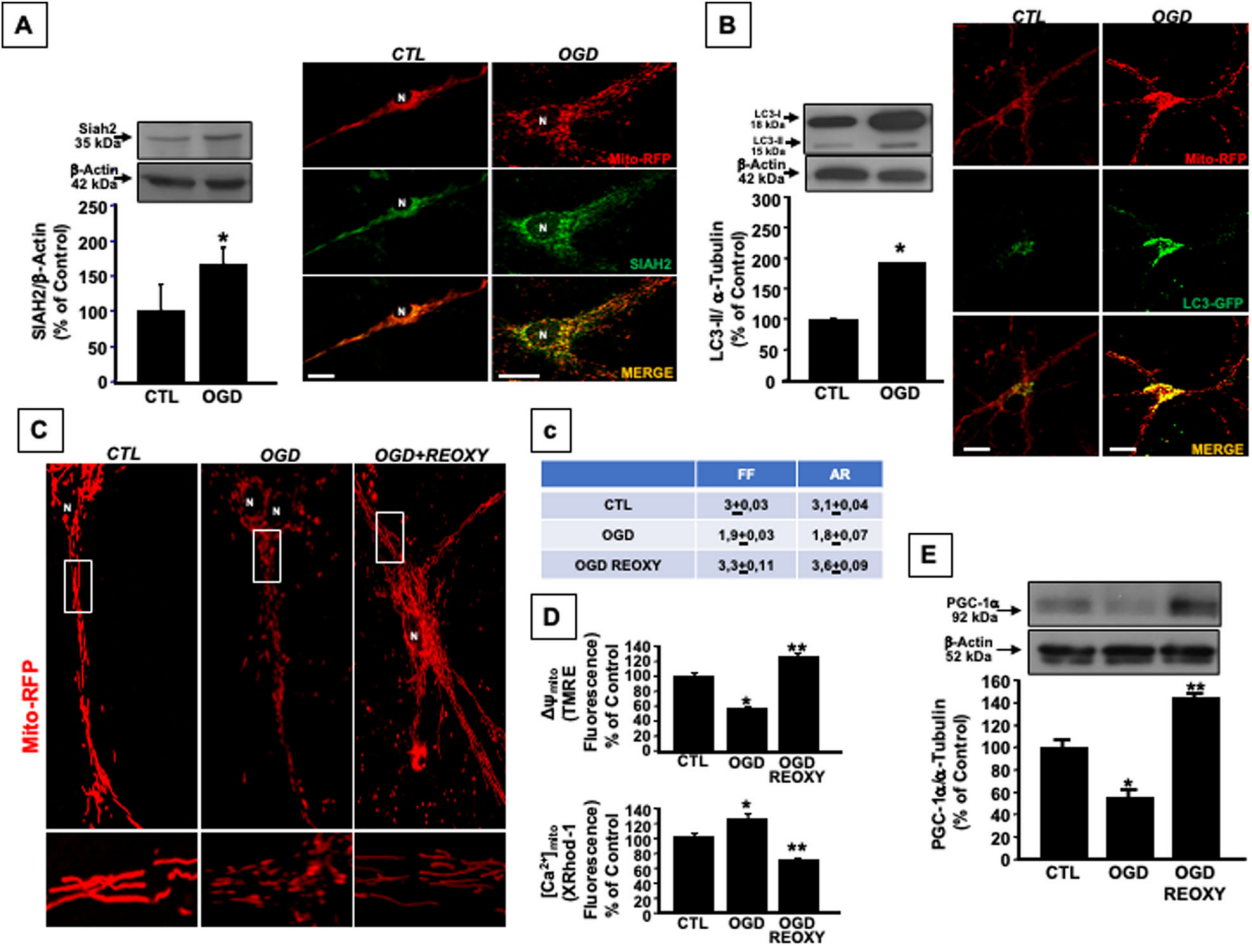
Mitochondrial dynamics in cortical neurons exposed to OGD and OGD/REOXY. **A** Left panel: Siah2 protein expression in cortical neurons exposed to OGD 3 h. Data are expressed as percentage ± SEM compared to CTL. *P < 0.05 vs CTL. Right panel: representative cortical neuron double-labeled with Siah2 (green) and Mito-RFP (red). Siah2/Mito-RFP colocalization was 100% in CTL condition and was up to 149% ± 3 after OGD 3 h. **B** Left panel: LC3-II protein expression in cortical neurons after 3 h OGD exposure. Each bar represents the mean±S.E.M. of the percentage of different experimental values obtained in three independent experimental sessions. *P < 0.05 vs CTL. Right panel: representative cortical neuron double-labeled with LC3-GFP (green) and Mito-RFP (red). LC3-GFP/Mito-RFP colocalization was 100% in CTL condition and was up to 161% ± 3 after 3 h of OGD. Bars 10 μM. **C** Mitochondrial morphology in cortical neurons labeled with Mito-RFP and exposed to 3 h OGD and OGD followed by 24 h reoxygenation (OGD/REOXY). The changes in mitochondrial morphology detectable in OGD and OGD/REOXY conditions have been quantified by using the ImageJ macro, “Morphometry” that allowed to determine two parameters FF indicative of the perimeter and area of a single mitochondria, and the AR representing the shape of linked, branched, or highly interconnected mitochondria. Low values of FF and AR indicate circular mitochondria, suggestive of mitochondrial fission, whereas high values indicate elongated and highly interconnected mitochondria, suggestive of mitochondrial fusion; **c** Quantification of the changes in mitochondrial morphology expressed in terms of FF and AR: FF: CTL: 3, OGD: 1,9, OGD/REOXY: 3,3; AR CTL: 3,1, OGD: 1,8, OGD RX: 3,6. **D** ΔΨ_m_ and [Ca^2+^]_m_ measurement in cortical neurons exposed to OGD and OGD/REOXY. ΔΨ_m_ and [Ca^2+^]_m_ were measured at the end of OGD or OGD/REOXY by using the fluorescent probes TMRE and X-Rhod1 and confocal microscopy, with the Bars 10 μM. Each bar represents the mean±S.E.M of the percentage of different experimental values obtained in three independent experimental sessions. *P < 0.05 vs CTL; **P < 0.05 vs CTL and OGD. **E** PGC-1α proteins expression in cortical neurons exposed to OGD and OGD/REOXY. Each bar represents the mean±S.E.M of the percentage of different experimental values obtained in three independent experimental sessions. *P < 0.05 vs CTL; **P < 0.05 vs CTL and OGD.

Similarly, when cortical neurons are exposed to 3 h of oxygen and glucose deprivation (OGD), an experimental condition mimicking in vitro the ischemic insult occurring in vivo, the mitochondrial membrane depolarization occurring [13, 18, 27] stimulates Siah2 protein expression and promotes its localization on mitochondria, as confirmed by immunocytochemistry experiments (Fig. 2A). This effect is accompanied by an increase in LC3-II protein expression, a marker of mitophagy, and by a massive colocalization between LC3-GFP and Mito-RFP immunosignal (Fig. 2B), thus indicating that mitochondrial dysfunction occurring during OGD causes the activation of Siah2-mediated mitophagy. However, the exposure of neurons to OGD followed by Reoxygenation (OGD/REOXY), determined an improvement in aspect ratio (AR) and form factor (FF), two parameters related to mitochondrial morphology, an increase of the ΔΨ_m_, and a reduction of mitochondrial calcium content ([Ca^2+^]_m_) (Fig. 2C, D). Indeed, when cortical neurons undergo OGD, the values of FF and AR are lower compared to those observed in control conditions, indicating that during OGD, mitochondria assume a circular shape, suggestive of mitochondrial fission. Conversely, when neurons undergo OGD/REOXY the values of FF and AR are higher compared to those observed in neurons exposed to OGD, indicating that during reoxygenation, mitochondria appear elongated and highly interconnected, suggestive of mitochondrial fusion (Fig. 2c).

These data let to hypothesize an increased clearance of mitochondria observed during OGD that may be the trigger for the activation of mitochondrial biogenesis in the survived neurons. To verify this hypothesis, the protein expression of PGC-1α, the master regulator of mitochondrial biogenesis, was investigated. As shown in Fig. 2E, an increase PGC-1α expression occurred during the reoxygenation phase.

### Siah2 and LC3-increased mitophagy and biogenesis in cortical neurons exposed to IPC leads to neuroprotection in the subsequent reoxygenation phase

**Fig. 3:**
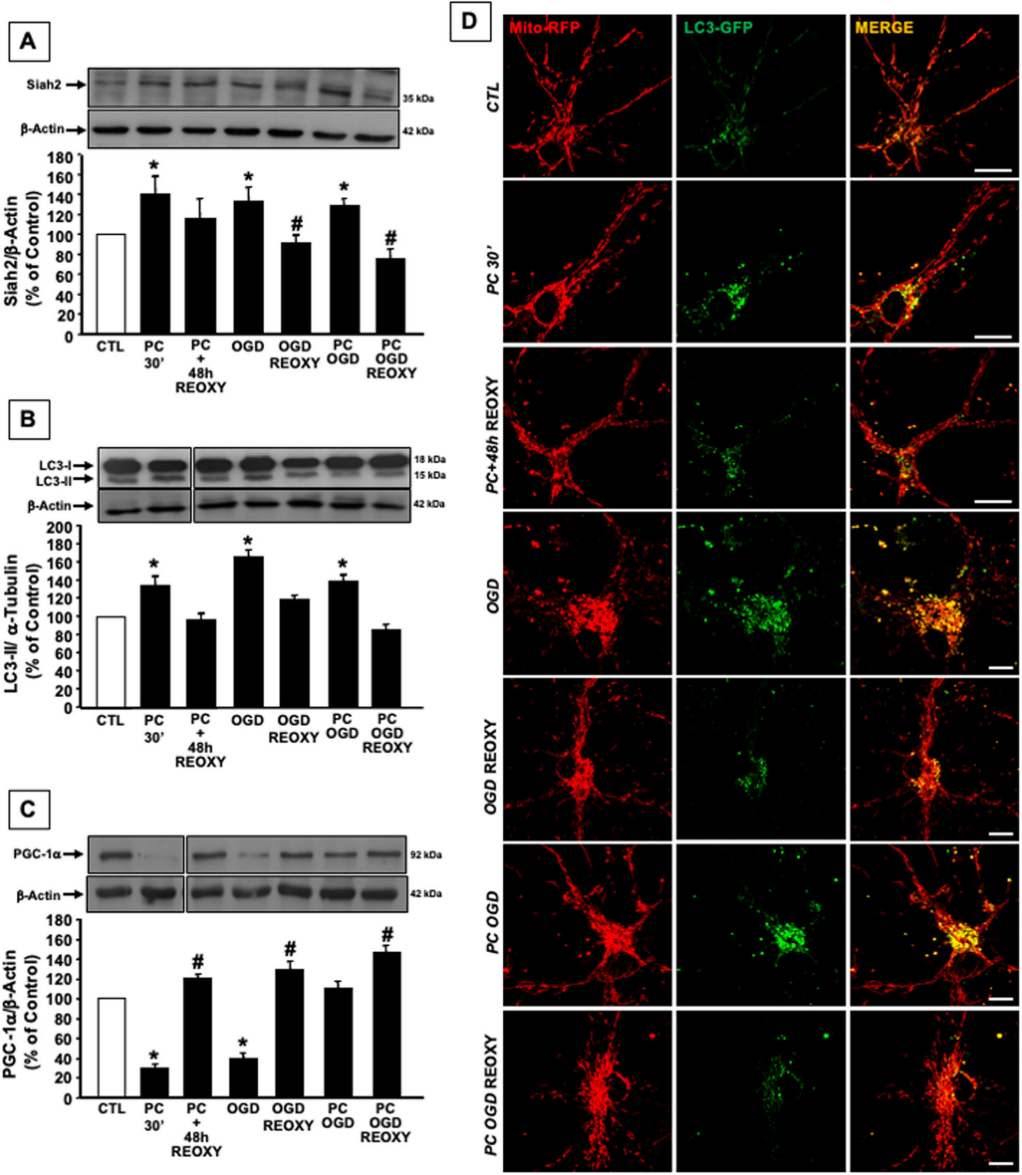
Mitophagy and mitochondrial biogenesis in preconditioned neurons exposed to OGD and OGD/REOXY. **A** Siah2, **B** LC3-II, **C** PGC-1α proteins expression measured by western blot analysis in cortical neurons exposed to IPC and IPC followed by OGD/REOXY. Each bar represents the mean ± S.E.M. of the percentage of different experimental values obtained in three independent experimental sessions. *P < 0.05 vs CTL; ^#^*P* < 0.05 vs its relative ischemic stimulus. **D** Colocalization between LC3-GFP (green) and Mito-RFP (red) in cortical neurons exposed to IPC and IPC followed by OGD/REOXY. LC3-GFP/Mito-RFP colocalization was 100% in CTL condition, 154% ± 1 after 30 min IPC, 112% ± 2 after IPC + 48 h RX, 173% ± 1 after 3 h of OGD, 124% ± 3 after OGD/REOXY, 161% ± 3 after 30 min IPC + OGD, 107% ± 3 after 30 min IPC + OGD RX. Bars 10 μM.

In order to understand whether the activation of mitophagy, observed in ischemic neurons, and the consequent mitochondrial biogenesis, elicited by the reoxygenation phase, might play a neuroprotective effect, further experiments have been performed in vitro in a widely used model of neuroprotection represented by ischemic brain preconditioning (IPC). As reported in Fig. 3A, B, the exposure of cortical neurons to 30 min of OGD, a condition comparable to a sublethal ischemic insult in vivo, increased Siah2 and LC3-II protein expression also when IPC is followed by 3 h of OGD, whereas the expression of these two proteins returns to the basal level during the reoxygenation phase (Fig. 3A, B). Similarly, immunocytochemistry experiments performed in cortical neurons co-transfected with Mito-RFP and LC3-GFP demonstrate an increase of the colocalization between LC3 and mitochondria in neurons exposed to preconditioning and in preconditioned neurons exposed to OGD, an effect prevented by the reoxygenation (Fig. 3D). Interestingly, in the above mentioned experimental conditions, PGC-1α expression is strongly decreased after 30 min and 3 h of OGD, whereas its expression increases in preconditioned neurons exposed to OGD and to OGD followed by reoxygenation (Fig. 3C).

**Fig. 4:**
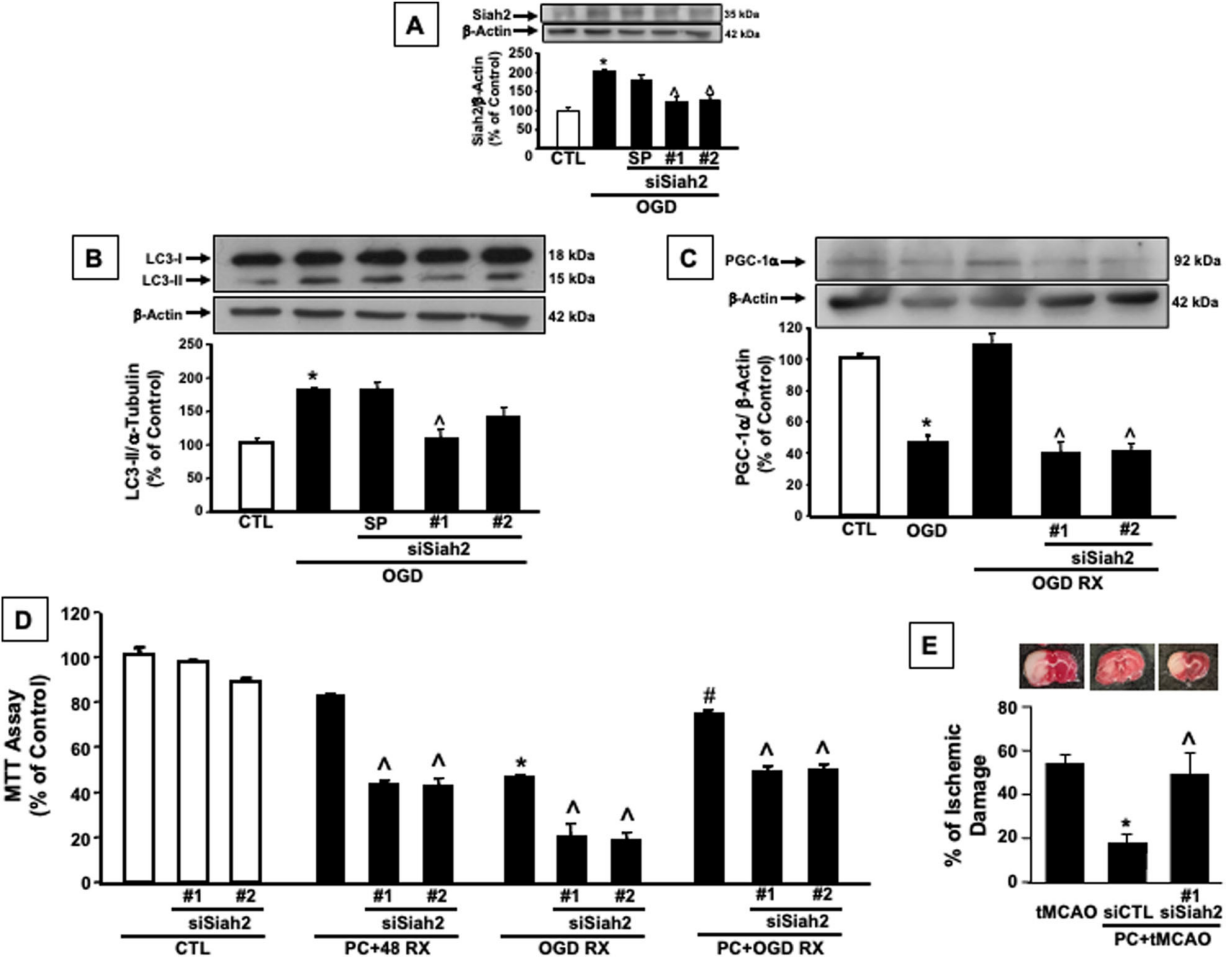
Siah2 activation is responsible for the IPC-neuroprotective effect. **A** Siah2 protein expression in siSiah2 (SMARTpool (SP), #1 and #2) transiently transfected cortical neurons exposed to OGD 3 h. **B** LC3-II protein expression in siSiah2 (SMARTpool (SP), #1 and #2) transiently transfected cortical neurons exposed to OGD 3 h. **C** PGC-1α protein expression in siSiah2 (#1 and #2) transiently transfected cortical neurons exposed to OGD 3 h and OGD/REOXY. **D** Mitochondrial function measured by MTT in siSiah2 (#1 and #2) transiently transfected cortical neurons exposed to IPC and IPC followed by OGD/REOXY. **E** Evaluation of ischemic damage in the ipsilateral temporoparietal cortex of siSiah2#1 rats after tMCAO and preconditioning+tMCAO followed by 24 h of reperfusion. Each bar represents the mean + S.E.M. of the percentage of different experimental values obtained in three independent experimental sessions. *P < 0.05 vs CTL; ^#^*P* < 0.05 vs OGD/REOXY; ^*P* < 0.05 vs its relative siCTL.

To further confirm the role of Siah2 in the activation of mitophagic machinery during ischemia and the consequent induction of mitochondrial biogenesis in the reoxygenation phase, small Siah2 interfering RNAs (siRNA) targeting distinct segments of Siah2 were transiently transfected in cortical neurons before OGD exposure. As shown in Fig. 4A, siRNASiah2 #1 or siRNASiah2 #2 significantly reduce OGD-mediated Siah2 activation and, interestingly siRNASiah2 #1 is also able to abrogate the conversion of LC3-I into its autophagy-related form LC3-II (Fig. 4B). Conversely, both siRNASiah2 #1 and #2 are able to prevent the increase of PGC-1α protein expression observed after the reoxygenation (Fig. 4C). These findings further support the role of Siah2 in the regulation of the balance between mitophagy and mitochondrial biogenesis in ischemic conditions. Therefore, to demonstrate that Siah2 ablation is capable of preventing IPC-induced neuroprotection, neuronal viability measured as mitochondrial oxidative capacity has been evaluated in cortical neurons exposed to IPC after the Siah2 silencing. In this experimental condition mitochondrial function is impaired, and the neuroprotective effect of IPC in cortical neurons exposed to a subsequent OGD/REOXY stimulus, is greatly prevented (Fig. 4D). In vivo experiments performed in siCtrl and siSiah2 intracerebroventricularly (ICV) perfused rats subjected to IPC followed by transiently middle cerebral artery occlusion (tMCAO) supports the in vitro obtained results. Indeed, as reported in Fig. 4E the ICV treatment with siRNASiah2 induces the loss of the neuroprotective effect mediated by preconditioning compared to mice exposed to tMCAO alone (% infarct volume: 55 ± 4 in mice subjected to tMCAO; 18.5 ± 3.8 in siCtrl subjected to preconditioning + tMCAO, versus 50.3 ± 9.3 in siSiah2 subjected to preconditioning + tMCAO).

## Discussion

The results of the present study demonstrated that the E3-ubiquitin ligase Siah2 plays a key role in neuroprotection induced by IPC by orchestrating the balance between mitophagy and mitochondrial biogenesis in cortical neurons exposed to OGD/Reoxygenation. Indeed, Siah2, activated by experimental conditions causing mitochondrial depolarization such as FCCP treatment or OGD exposure, was able to promote LC3 conversion from its cytosolic form LC3-I into its autophagy-related form LC3-II, an effect occurring in mitochondria as suggested by immunohistochemistry experiments. These effects were associated with changes in mitochondrial morphology and mitochondrial calcium content and were in line with our previous results reported in cortical neurons exposed to hypoxic conditions [18]. Interestingly, in the present study, when cortical neurons were exposed to OGD/REOXY, a condition associated with mitochondria hyperpolarization and reduction in mitochondrial calcium content, the activation of mitophagy was followed by an increase in PGC-1α protein expression, suggestive of the mitochondrial biogenesis activation in the reoxygenation phase. This effect might represent the attempt of neurons to activate intracellular mechanisms aimed at preserving cellular metabolism in stressful conditions. Indeed, the expression levels of proteins involved in the regulation of mitochondrial functions return to the basal values and mitochondria appear partially dysfunctional and morphologically elongated [18, 41, 48], suggestive of an imbalance between fission and fusion events. However, this finding, apparently controversial, further supports the hypothesis that the tight relationship existing between mitochondrial dysfunction and their morphology is extremely important to activate the complex mechanisms of mitochondrial quality control systems aimed at preserving cellular survival in stressful conditions [18, 49]. Accordingly, the results reported in the present study regarding mitochondrial calcium concentrations and membrane potential, associated with elongated/fused mitochondria and increased expression of PGC-1α, as detected in neurons during reoxygenation, greatly suggest that the improvement in mitochondrial morphology and function might be correlated to the activation of mitochondrial biogenesis.

Moreover, the results obtained in preconditioned neurons exposed to OGD and OGD followed by reoxygenation further reinforced this suggestion and confirmed that mitochondria exert a pivotal role in neuroprotection mediated by IPC [34]. As reported in the present study, the increase in mitophagy, observed in cortical neurons exposed to IPC or OGD and testified by LC3-II increased expression, was counterbalanced by the activation of mitochondrial biogenesis in preconditioned neurons after reoxygenation as confirmed by the increase in PGC-1α protein expression. The new finding emerging by the present work was that all the effects above described were regulated by Siah2 protein activation during ischemic conditions, since the treatment with siSiah2 completely prevented the activation of those intracellular events culminating in mitochondrial quality control activation i.e. mitophagy and mitochondrial biogenesis, and abolished IPC-induced neuroprotection both in in vitro and in in vivo models of ischemic tolerance. On the other hand, Siah2 played a key role in the preservation of mitochondrial function during anoxia was also revealed in siah2−/− mice [18]. Indeed, Siah2 ablation, by preventing the OGD-induced degradation of mitochondrial proteins involved in the regulation of calcium homeostasis like AKAP121 and NCX3, was able to preserve the balance between fragmentation and fusion, two events belonging to the complex mechanisms of mitochondrial quality control, and to counteract increases in [Ca^2+^]_m_ and mitochondrial depolarization. Moreover, experiments performed in neurons obtained from ncx3−/− mice, showing higher calcium levels and fragmentation in ncx3−/− neurons than in ncx3+/+ neurons, further support the critical role of NCX3 and Siah2 in regulating mitochondrial function and dynamics in hypoxic conditions [18]. These findings correlate with data reported in the present study, demonstrating that hypoxic-induced Siah2 activation promotes mitochondrial protein ubiquitination, stimulating LC3-induced mitophagy. Therefore, it is possible to speculate that Siah2 induction during IPC altered mitochondria morphology and function thus promoting the removal of dysfunctional mitochondria by mitophagy and activating mitochondrial biogenesis to restore cellular metabolism in stressful conditions. Indeed, preconditioning positively affects the integrity of mitochondrial oxidative phosphorylation after cerebral ischemia, prevents mitochondrial swelling, protects mitochondrial energy metabolism during cerebral ischemia by avoiding ATP consumption [29], and increases Mn-SOD expression and activity through the NO/Ras/ERK1-2 pathway [33]. In this regard, Siah2 might work as a sensor of mitochondrial dysfunction in hypoxic conditions, thus activating the mitochondrial quality control process, as already described for the E3-ubiquitin ligase Parkin in Parkinson’s Disease [37].

Taken together, these results allow us to conclude that Siah2-dependent mitophagy activation during the ischemic insult stimulates mitochondrial biogenesis in the reoxygenation phase to provide energy metabolism required for neuronal survival after ischemia. The balance between mitophagy and mitochondrial biogenesis is crucial for neuroprotection induced by IPC. Interestingly, the genetic ablation of Siah2 by siRNA administration in preconditioned mice exposed to ischemia was able to counteract IPC-induced neuroprotection. In this scenario, Siah2 might represent a molecular druggable target useful for the development of new therapeutic strategies for ischemia.

## Materials and methods

### Primary cortical neurons

#### Post-natal neurons

Cultures of cortical neurons from Wistar rat pups, 2–4 days old, were prepared as previously described [50]. Cells were plated at 1.8 × 10^6^ on 25-mm glass coverslips pre-coated with poly-D-lysine (10 mg/ml) and used for confocal microscopy analysis.

#### Embryonic neurons

Cortical pure neurons were prepared from the brains of 16-day-old Wistar rat embryos, as already described [51]. Cells were plated at 1.5 × 10^6^ in 12-wells dishes or at 15 × 10^6^ in 100-mm plastic Petri dishes. Ara-C (10 μM) was added 48 after plating to stop non-neuronal cell growth. All neuronal cells were cultured at 37 °C in a humidified 5% CO2 atmosphere and used after 10 days of culture, for western blot analysis and diphenyltetrazolium bromide (MTT) experiments.

The reason to use embryonic and post-natal primary neurons was dictated only by technical aspects since the post-natal neurons grow better on glass compared to the embryonic neurons. No differences in terms of metabolic and functional properties between the two different neuronal preparations have been reported. All the experiments on primary cortical neurons were performed according to the procedures described in experimental protocols approved by the Ethical Committee of the ‘Federico II’ University of Naples.

### Oxygen and glucose deprivation

IPC and OGD insults were reproduced in cortical neurons by exposing cells to a medium deprived of serum, oxygen, and glucose for 30 min and 3 h, respectively, to mimic in vitro a condition similar to the in vivo ischemia, as previously reported [34, 52]. These ischemic conditions were maintained using a hypoxia chamber (Billups Rothemberg Inc. Del Mar.) (temperature 37 °C, under an atmosphere of 5% CO2 and 95% N2). Bafilomycin at [1 μM]_f_, was added to the medium deprived of oxygen, serum, and glucose, during the IPC and OGD phases to detect LC3 protein expression. Reoxygenation was obtained by incubating the cells for 24 h in the presence of normal levels of serum, glucose, and oxygen.

### Transient focal ischemia and IPC

Transient focal ischemia was induced in male adult Sprague Dawley rats under an operating stereomicroscope (Nikon SMZ800, Nikon Instruments, Florence, Italy), by middle cerebral artery occlusion. The right cerebral artery was occluded for 30 min for preconditioning and 100 min for ischemia. Achievement of ischemia was confirmed by monitoring the cerebral blood flow (CBF) through a disposable micro-tip fiber optic probe (diameter 0.5 mm) (PF5001; Perimed Järfälla, Sweden) [53]. Animals that did not show a CBF reduction of at least 70% or die after the surgical procedure were excluded. All experiments were performed according to the Guide for the Care and Use of Laboratory Animals and approved by the Animal Care Committee of “Federico II”, University of Naples, Italy. Every attempt was made to reduce the number of animals used and to minimize animal suffering. Animals were randomly allocated to each experimental group by using the method of simple randomization.

### Intracerebroventricular administration

Rats previously anesthetized with sevoflurane were positioned on a stereotaxic frame, a 23-g stainless steel guide cannula was implanted into the right lateral ventricle using the stereotaxic coordinates from the bregma: 0.4 mm caudal, 2 mm lateral and 2 mm below the dura. A surgically implanted steel cannula was used to administer the negative siCtl or siSiah2. 10 μl of siRNAs, administered at the beginning and after 3 h of the reperfusion from a [10 μM]_f_ stock, were injected directly into the right lateral ventricle slowly through the connection between a syringe suitably loaded with the siRNA connected to the tube that reaches the stainless steel of the cannula [54].

### Evaluation of the infarct volume

For the analysis of ischemic damage, rats were euthanized 24 or 72 h after preconditioning+tMCAO. The ischemic volume was evaluated with 2,3,5-triphenyl tetrazolium chloride staining and calculated with image analysis software (Image-ProPlus). The total infarct volume was expressed as percentage of the volume of the hemisphere ipsilateral to the lesion to correct brain edema and, evaluated in a blind manner [55].

### Mitochondrial extracts

#### Extraction with differential centrifugation

Mitochondria were isolated from cortical neurons by differential centrifugation as previously described [13]. By this protocol, three different fractions were obtained corresponding to the membrane, cytosol, and mitochondria compartments. After the first centrifugation, pellets, corresponding to the fraction containing membranes but not intracellular organelles, including mitochondria, were separated from supernatants and measured for protein concentrations. The supernatant was centrifuged at 500 × *g* for 5 minutes at 4 °C, and the supernatant obtained corresponding to the cytosolic fraction containing the organelles was further centrifuged at 19,000 × *g* for 10 minutes at 4 °C to separate the mitochondrial from the cytosolic fraction. Supernatants (Cytosol) were then removed and evaluated for protein content. Next, the pellets containing mitochondria were lysed in 50 μl of lysis buffer, purified again by centrifugation (18,000 × *g*, 10 minutes), and supernatants (Mitochondria) were assessed for protein content by Bradford’s assay [56]. The fractions obtained were used for western blotting analysis. The purity of the mitochondrial preparation was assessed in a previous paper by our group [13].

### Western blot

Proteins samples (50 μg) were separated on 10% sodium dodecyl sulfate-polyacrylamide gels for Siah2, PGC-1α, VDAC, and 15% for LC3 and electrotransferred onto Hybond ECL nitrocellulose paper (Amersham, Milan, Italy) [18]. Membranes were incubated overnight at 4 °C in the blocked buffer with the 1:1000 antibody for: Siah2 (polyclonal rabbit antibody, abcam), PGC-1α (polyclonal rabbit antibody, Abcam), LC3 (polyclonal rabbit antibody, Sigma-Aldrich), VDAC (monoclonal mouse antibody, Millipore), α-tubulin (1:5000 mouse monoclonal antibody, Sigma-Aldrich) and β-actin (Anti-β-Actin peroxidase conjugate, Sigma-Aldrich). The optical density of the bands was determined by the Chemi Doc Imaging System (Bio-Rad, Milan, Italy).

Full and uncropped western blots are reported as supplemental material.

### Immunocytochemistry

Neurons were rinsed twice in cold 0.01 M PBS at pH 7.4 and fixed at room temperature in 4% (w/v) paraformaldehyde for 20 minutes. Following three washes in PBS, cells were blocked in PBS containing 3% BSA, and the following antibody anti-Siah2 1:200 (polyclonal rabbit antibody, abcam) was then incubated overnight at 4 °C. Next, slides were washed in PBS, incubated with anti-rabbit Cy2-conjugated antibody (Jackson, dilution 1:200) for 1 h at room temperature under dark conditions, and washed again with PBS. Finally, they were mounted with a SlowFadeTM Antifade Kit (Molecular Probes-Invitrogen) and analyzed by confocal microscopy [35]. Mitochondria were labeled by incubating cells with MitoTracker Red (Invitrogen, 20 nM) for 20 min prior to acquisition or transfecting cells with Mito-RFP. LC3 immunosignal was detected by using a construct encoding for LC3-GFP. Cells were analyzed for colocalizations: a) Mito (red) and Siah2 (green) by using the ‘colocalization highlighter’ plug-in for ImageJ Software (NIH, Bethesda, MA, USA); b) Mito-RFP and LC3-GFP by using a custom-written ImageJ macro containing plug-ins described by Dagda and Chu [57, 58]. Before colocalization analysis, threshold settings for each image were determined, and quantification was achieved by counting the number of Siah2/Mito or LC3/Mito colocalized points per microscope field. Results were expressed as a percentage of colocalization [13].

### Analysis and quantification of changes in mitochondrial morphology using the IMAGEJ 1.42 software

Mitochondria were labeled by incubating cells with MitoTracker Red (Invitrogen, 20 nM) for 20 min prior to acquisition or transfecting cells with Mito-RFP. Digital images were captured with a confocal microscope as previously described [18]. Mitochondrial shape metrics were analyzed by using the ImageJ macro, “Morphometry” [59] that allowed to determine two parameters of mitochondrial morphology: FF and AR. The former takes into account the perimeter and area of a single mitochondrion and can, therefore, capture complex mitochondrial shapes. The latter, instead, despite being a useful shape metric for simple rod-like mitochondria, does not faithfully represent the shape of linked, branched, or highly interconnected mitochondria. Low values of FF and AR indicate circular mitochondria, suggestive of mitochondrial fission, whereas high values indicate elongated and highly interconnected mitochondria, suggestive of mitochondrial fusion [59].

### Imaging of mitochondrial Ca^2+^ and mitochondrial membrane potential

[Ca^2+^]_m_ and mitochondrial membrane potential were assessed by using the fluorescent dyes X-Rhod1 and TMRE, respectively, as described by Sisalli et al. [18]. Confocal images were obtained with a Zeiss inverted 700 confocal laser scanning microscopy and a ×63 oil immersion objective. The illumination intensity of 543 Xenon laser, used to excite X-Rhod-1 and TMRE fluorescence, was kept to a minimum of 0.5% of laser output to avoid phototoxicity.

### MTT assay

Mitochondrial activity was assessed by the 3-(4,5-dimethylthiazol-2-yl)-2,5, MTT assay, as previously described [34, 51]. The assay was based on the redox ability of living mitochondria to convert dissolved MTT into insoluble formazan. The absorbance was monitored at 540 nm with a Perkin-Elmer LS 55 luminescence spectrometer (Perkin-Elmer Ltd., Beaconsfield, England). The data are expressed as a percentage of cell damage compared with control neurons.

### Plasmids and transfection

pMYs-IRES-mito-RFP was a gift from Qing Deng (Addgene plasmid # 121996) [60] and EGFP-LC3 was a gift from Karla Kirkegaard (Addgene plasmid #11546) [61]. To knock down Siah2, siGENOME duplex siRNAs and siGENOME SMARTpool targeting four distinct segments of Siah2 were purchased from Dharmacon. Three different siRNASiah2 mixtures have been used: (a) siRNASiah2 SMARTpool, containing equimolar concentrations of all four duplex siRNAs, SP; (b) siRNASiah2 #1, containing equimolar concentrations of two duplex siRNAs (D-041993-01, D-041993-02) and (c) siRNASiah2 #2, containing equimolar concentrations of two duplex siRNAs (D-041993-03 and D-041993-04) [10]. All these constructs were transiently transfected using Lipofectamine 2000 (Invitrogen). The siRNAs were transiently transfected at a final concentration of 250 pmol/ml of culture medium. After 5 h, it was replaced with fresh medium [34].

### Materials

All the reagents were purchased from Sigma Chemicals (Milan, Italy) unless otherwise specified.

### Statistical analysis

Sample size for each experimental group has been calculated by G-power software. Data were generated from a minimum of three independent experiments. Ca^2+^ and ΔΨ_m_ measurements were performed at least in 20 cells for each of the three independent experimental sessions. Data were expressed as mean + S.E.M. Statistical analysis was performed with analysis of variance followed by Newman–Keuls test. Statistical significance was accepted at the 95% confidence level (*P* ≤ 0.05).

## Supplementary information

Uncropped original western blotting

## Data Availability

The authors confirm that the data supporting the findings of this study are available within the article [and/or] its supplemental information.
