# Supplementary material for: The E3-ligase Siah2 activates mitochondrial quality control in neurons to maintain energy metabolism during ischemic brain tolerance
Source: Cell Death Dis. 2025 Jan 28;16(1):52. doi: 10.1038/s41419-025-07339-z (PMC11775118; doi:10.1038/s41419-025-07339-z)

Fig 1 B

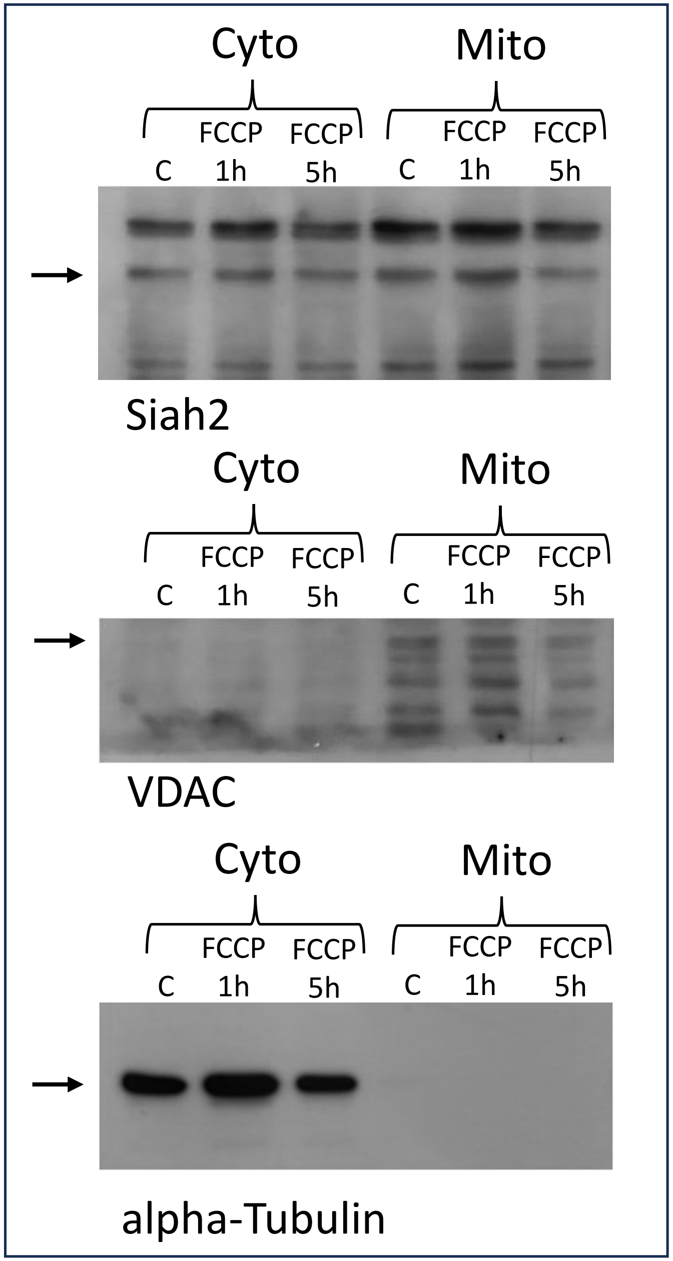

Fig 1 C

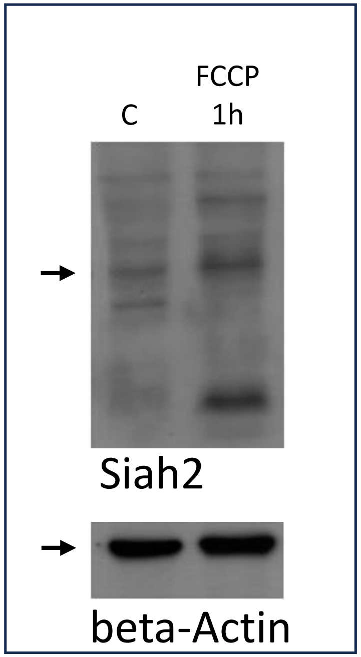

Fig 1 D

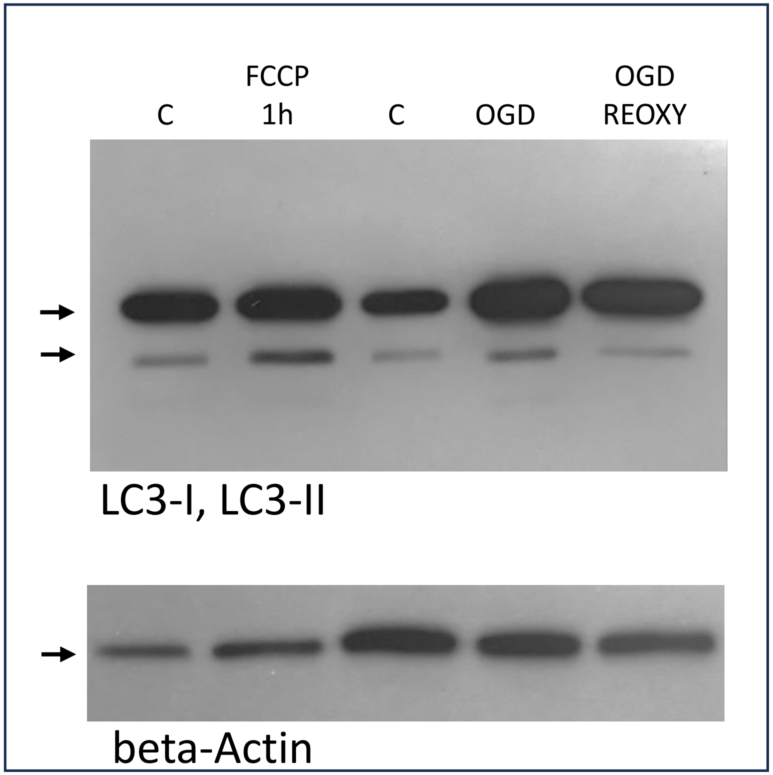

Fig 2 A

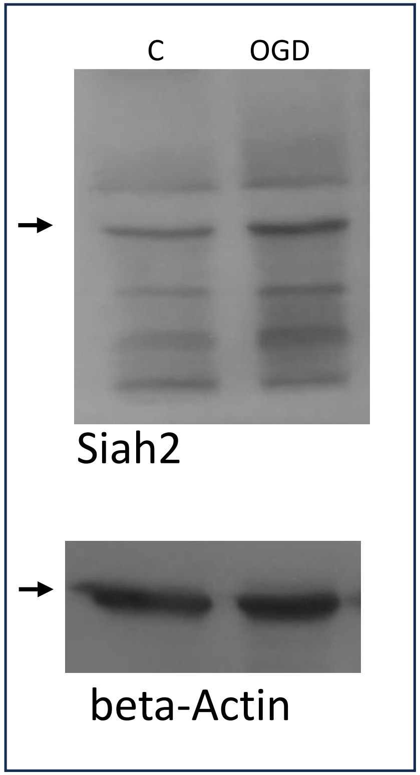

Fig 2 B

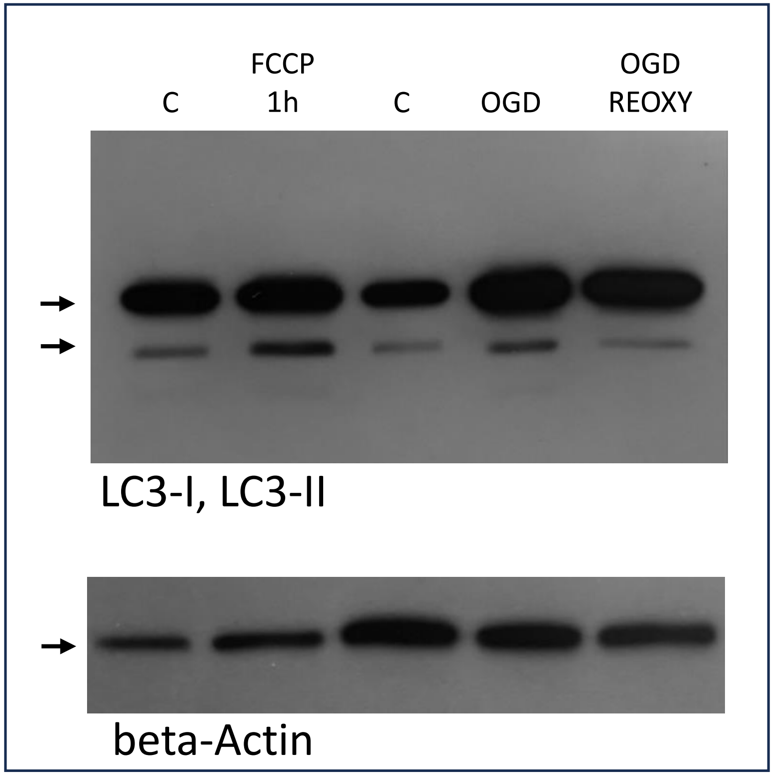

Fig 2 E

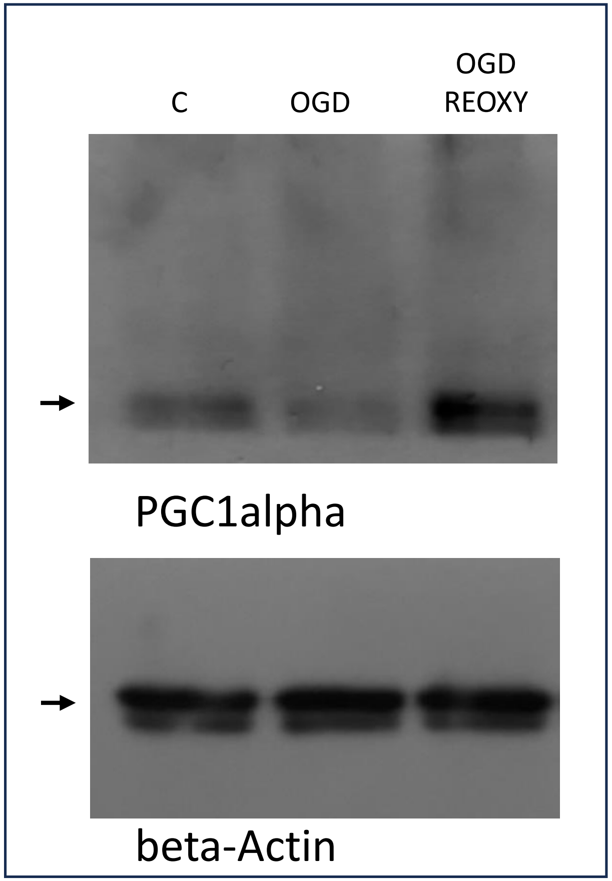

Fig 3A

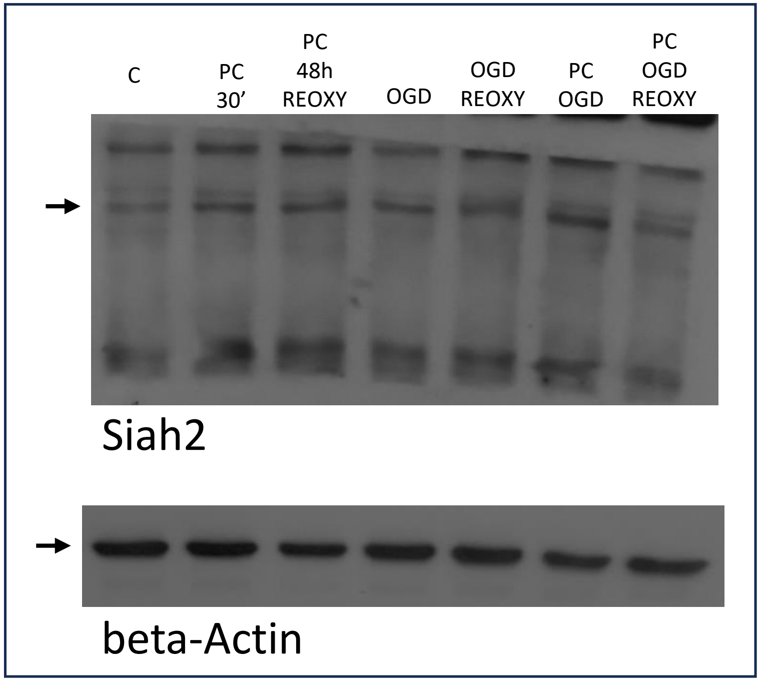

Fig 3B

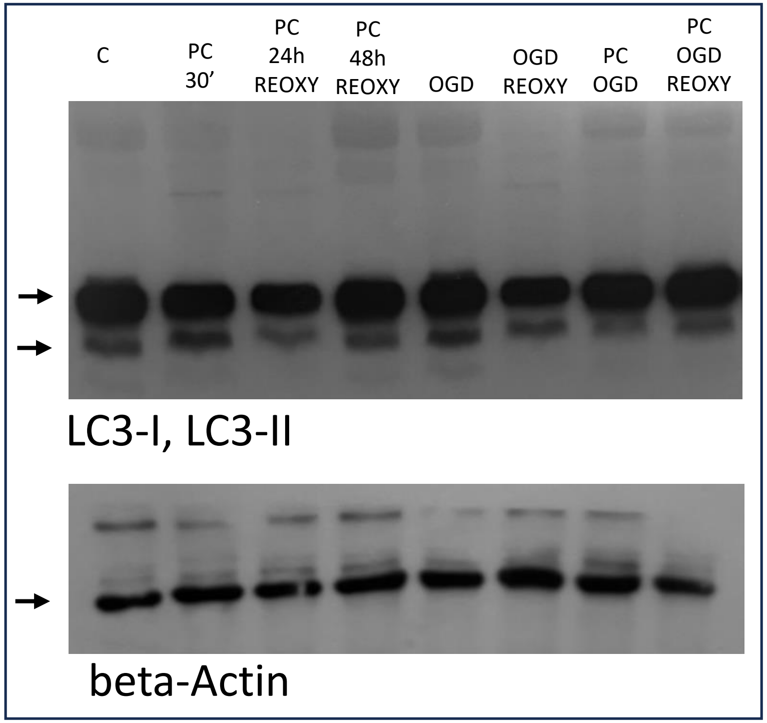

Fig 3C

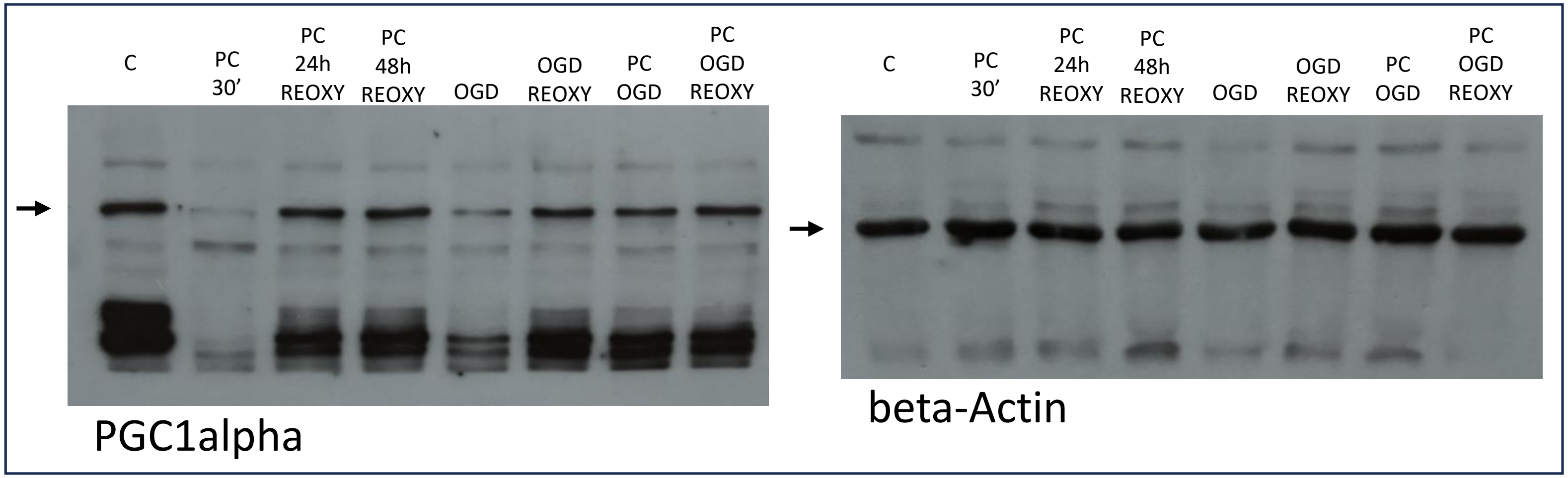

Fig 4A

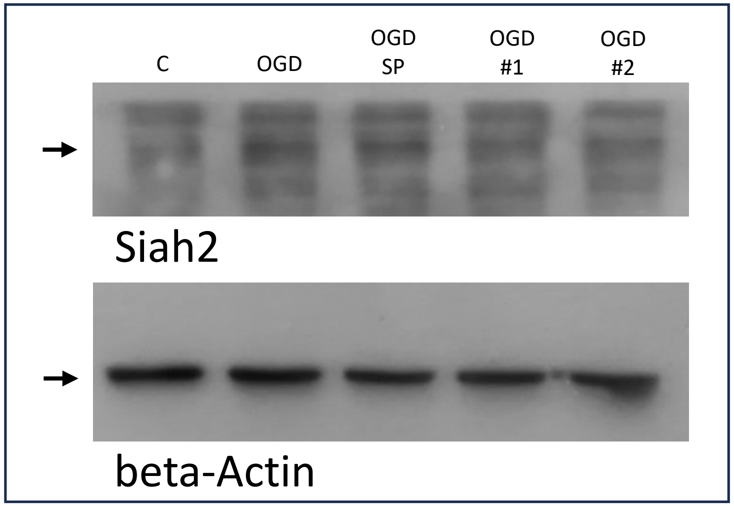

Fig 4B

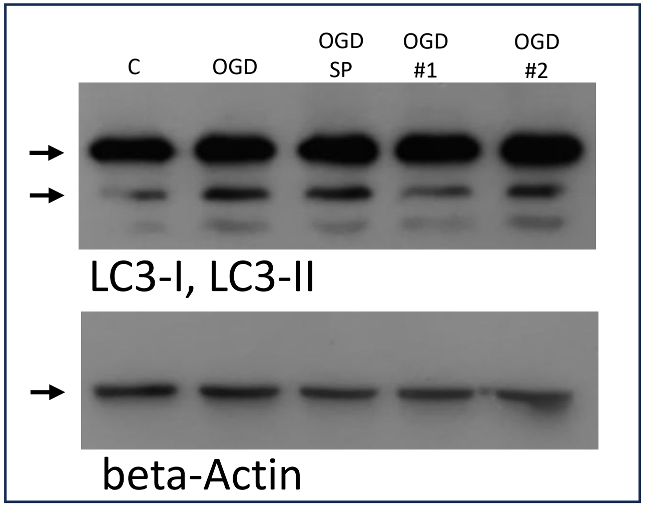

Fig 4C

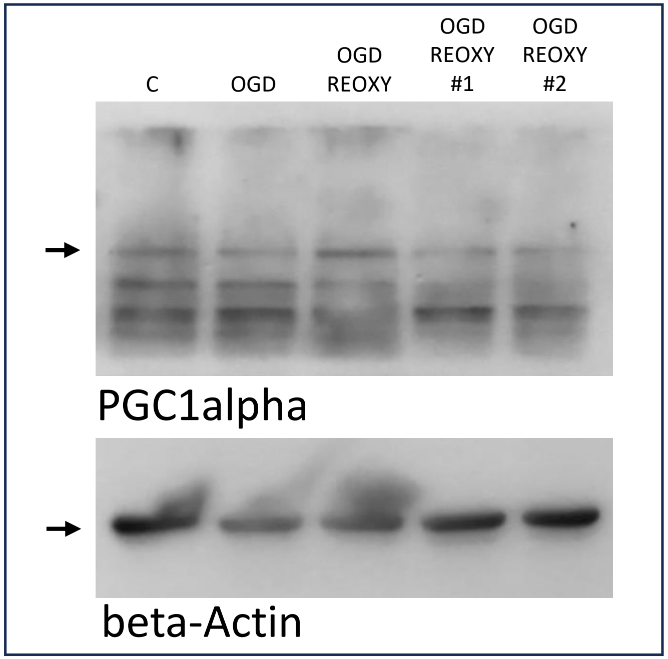

Supplement: Supplementary file 1 — Uncropped original western blotting [file 41419_2025_7339_MOESM1_ESM.pdf]
